# Assessing the Effects of Curcumin and 450 nm Photodynamic Therapy on Oxidative Metabolism and Cell Cycle in Head and Neck Squamous Cell Carcinoma: An In Vitro Study

**DOI:** 10.3390/cancers16091642

**Published:** 2024-04-24

**Authors:** Silvia Ravera, Claudio Pasquale, Isabella Panfoli, Matteo Bozzo, Dimitrios Agas, Silvia Bruno, Michael R. Hamblin, Andrea Amaroli

**Affiliations:** 1 Department of Experimental Medicine (DIMES), University of Genoa, 16132 Genoa, Italy; silvia.bruno@unige.it; 2 Department of Surgical and Diagnostic Sciences (DISC), University of Genoa, 16132 Genoa, Italy; clodent@gmail.com; 3 Department of Pharmacy (DIFAR), University of Genoa, 16132 Genoa, Italy; 4 BIO-Photonics Overarching Research Laboratory (BIOPHOR), Department of Earth, Environmental and Life Sciences (DISTAV), University of Genoa, 16132 Genoa, Italy; matteo.bozzo@unige.it (M.B.); andrea.amaroli@unige.it (A.A.); 5 School of Biosciences and Veterinary Medicine, University of Camerino, 62032 Camerino, Italy; dimitrios.agas@unicam.it; 6 Laser Research Centre, Faculty of Health Science, University of Johannesburg, Johannesburg 2092, South Africa; hamblin.lab@gmail.com

**Keywords:** photochemotherapy, head and neck squamous cell carcinoma (HNSCC), human primary fibroblasts, oxidative stress, oxidative phosphorylation (OxPhos) activity, energy metabolism, intracellular malondialdehyde concentration, antioxidant enzymes activity, cellular growth, viability

## Abstract

**Simple Summary:**

The study investigates the effects of curcumin in native conditions and after irradiation with 450 nm light on the energy metabolism, redox balance, and cellular growth of head and neck cancer cells and human primary fibroblasts. Although curcumin in native conditions already shows an anti-cancer effect, affecting energy metabolism and limiting the growth of tumor cells and cells that inhabit the surrounding microenvironment, irradiation with 450 nm light (photodynamic) enhances its effect by acting on antioxidant defenses. This study, therefore, opens up new perspectives on specific wavelengths of light that appear to be able to improve the drug’s action on cancer growth and proliferation, offering hope for better patient outcomes in the future.

**Abstract:**

Oral cancer is the 16th most common malignant tumor worldwide. The risk of recurrence and mortality is high, and the survival rate is low over the following five years. Recent studies have shown that curcumin causes apoptosis in tumor cells by affecting F_o_F_1_-ATP synthase (ATP synthase) activity, which, in turn, hinders cell energy production, leading to a loss of cell viability. Additionally, irradiation of curcumin within cells can intensify its detrimental effects on cancer cell viability and proliferation (photodynamic therapy). We treated the OHSU-974 cell line, a model for human head and neck squamous cell carcinoma (HNSCC), and primary human fibroblasts. The treatment involved a 1 h exposure of cells to 0.1, 1.0, and 10 μM curcumin, followed or not by irradiation or the addition of the same concentration of pre-irradiated curcumin. Both instances involved a diode laser with a wavelength of 450 nm (0.25 W, 15 J, 60 s, 1 cm^2^, continuous wave mode). The treatment with non-irradiated 1 and 10 µM curcumin caused ATP synthase inhibition and a consequent reduction in the oxygen consumption rate (OCR) and the ATP/AMP ratio, which was associated with a decrement in lipid peroxidation accumulation and a slight increase in glutathione reductase and catalase activity. By contrast, 60 s curcumin irradiation with 0.25 W—450 nm caused a further oxidative phosphorylation (OxPhos) metabolism impairment that induced an uncoupling between respiration and energy production, leading to increased oxidative damage, a cellular growth and viability reduction, and a cell cycle block in the G1 phase. These effects appeared to be more evident when the curcumin was irradiated after cell incubation. Since cells belonging to the HNSCC microenvironment support tumor development, curcumin’s effects have been analyzed on primary human fibroblasts, and a decrease in cell energy status has been observed with both irradiated and non-irradiated curcumin and an increase in oxidative lipid damage and a slowing of cell growth were observed when the curcumin was irradiated before or after cellular administration. Thus, although curcumin displays an anti-cancer role on OHSU-974 in its native form, photoactivation seems to enhance its effects, making it effective even at low dosages.

## 1. Introduction

Cancer remains a principal cause of death around the world, with the World Health Organization (WHO) reporting an alarming 19 million newly diagnosed cases in 2020 [1]. The 16th most common malignant tumor is oral cancer, which has a low 5-year survival rate and significant occurrences of death and recurrence [2].

Despite the use of multimodality treatment for head and neck squamous cell carcinoma (HNSCC), a significant proportion of patients show only a limited response to therapy. Currently, the standard approach for locally advanced HNSCC involves surgically removing the primary tumor along with affected lymph nodes, followed by radiotherapy, which is sometimes combined with platinum-based chemotherapy [1,3]. However, these treatment approaches often result in severe adverse effects that compromise the patient’s quality of life. Improving HNSCC therapy choices is crucial, particularly for recurring or metastatic cases. In this context, photodynamic therapy (PDT) has emerged as a promising alternative to meet this need [3,4,5], especially when used to enhance the anti-cancer effect of molecules with phototoxic properties such as curcumin, a natural polyphenolic substance [6]. In detail, curcumin possesses conjugated bonds acting as a π system, allowing it to absorb light with a broad absorption peak (300–500 nm). Curcumin has a maximum excitation wavelength of 425 nm and a maximum emission wavelength of 530 nm [7,8]. Therefore, it can be photoactivated, inducing potent phototoxic effects at micromolar concentrations [8] and increasing its detrimental activity on cancer cells [9]. On the other hand, curcumin contains a phenol group, a ketone group, and a methoxy group, which contribute to its anti-bacterial, anti-inflammatory, antioxidant, anti-angiogenic, antifungal, anti-proliferative, anti-invasion, and pro-apoptotic activities [8,10,11]. In addition, recent studies have demonstrated that curcumin affects F_o_F_1_-ATP synthase (ATP synthase) [12], impairing energy production and leading to the decreased viability of cancer cells through apoptosis [12,13].

In essence, PDT exerts its cytotoxic effects on pathogenic or undesired cells through the synergistic interaction of three components: a systemic or locally administered photosensitizing compound, light with the correct wavelength, and the surrounding oxygen [14,15,16]. The generation of singlet oxygen and other highly reactive oxygen species initiates a cascade of biochemical events that can ultimately lead to significant toxicity and trigger cell death through processes such as apoptosis or necrosis [15]. PDT has been successfully applied in microbiology, dermatology, gynecology, urology, ophthalmology, and oncology [14]. Moreover, a thorough examination of the current literature indicates that PDT has potential as a treatment approach for early-stage HNSCC. However, the limited number of studies suggests there may be certain limitations [5].

Therefore, herein, we examined how curcumin affects the OHSU-974 cell line, a human HNSCC model, and primary human fibroblasts as they play a pivotal role in the head–neck tumor microenvironment [17,18]. We evaluated the effects of curcumin, in its native form or after irradiation with a 450 nm wavelength diode laser, both before and after administering it. We assessed the ability of both native and irradiated curcumin to modulate the energetic metabolism and oxidative stress by analyzing the oxidative phosphorylation (OxPhos) activity and efficiency, energy status, the intracellular malondialdehyde concentration (a marker of lipid peroxidation), the activation of enzymatic antioxidant defenses, cellular growth and viability, and the cell cycle. In addition, as the chosen laser parameters could also display a photobiomodulation effect through a photon energy transfer to the cell, modulating cell metabolism and homeostasis without causing significant thermal increases, we also investigated the potential effect of the nm wavelength diode laser alone on the OHSU-974 cell line.

## 2. Materials and Methods

### 2.1. Cell Lines

The OHSU-974 cell line was generously provided by Prof. Susanne Wells at Cincinnati Children’s Hospital Medical Center in Cincinnati, OH, USA, and served as a human HNSCC model. Due to its origin in patients affected by Fanconi anemia, the OHSU-974 cell line underwent correction with S11FAIN retrovirus to eliminate any bias resulting from the FANC-A gene mutation [19]. OHSU-974 cells and primary fibroblast were cultured in DMEM (#61965026, Invitrogen, Waltham, MA, USA) supplemented with 10% fetal calf serum (#ECS0160L, Euroclone, Pero (MI), Italy) at 37 °C with 5% CO_2_ [20].

### 2.2. Experimental Setup, Curcumin Treatments, and Irradiated Parameters

**Figure 1:**
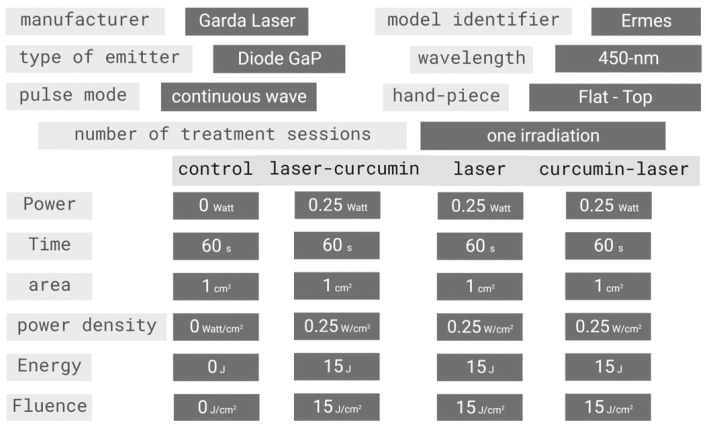
Device specifications and irradiation parameters.

The irradiation was conducted using a 450 nm wavelength diode laser (Garda Laser snc, Verona, Italy), with the specifications given in Figure 1. Photons were delivered through a flat-top profiled handpiece covering a 1 cm^2^ area, which encompassed the entire surface of the well in which the cells were cultured. As detailed in our previous reports [21,22], the flat profile of the handpiece ensured uniform energy delivery onto the irradiated surface, regardless of the distance from the target. However, to standardize the experimental setup, the irradiations were still carried out with the handpiece attached to a stand and positioned to be in contact with the multiwell culture plate.

All irradiations were conducted by placing the multiwell plates on a photon-absorbing sheet to eliminate any reflection from the top of the workbench (Metal Velvet light-absorbing panel, Acktar Ltd., Kiryat-Gat, Israel). Before irradiation, the correlation between the power settings on the laser device and the delivered power was assessed using a Pronto-250 power meter (Gentec Electro-Optics, Inc., G2E Quebec City, QC, Canada). The temperature in the irradiated wells was monitored during delivery using a FLIR ONE Pro-iOS thermal camera (resolution 0.1 °C, dynamic range: +400 °C/−20 °C; FLIR Systems, Inc., Portland, OR, USA). The irradiation parameters are specified in Figure 1.

**Figure 2:**
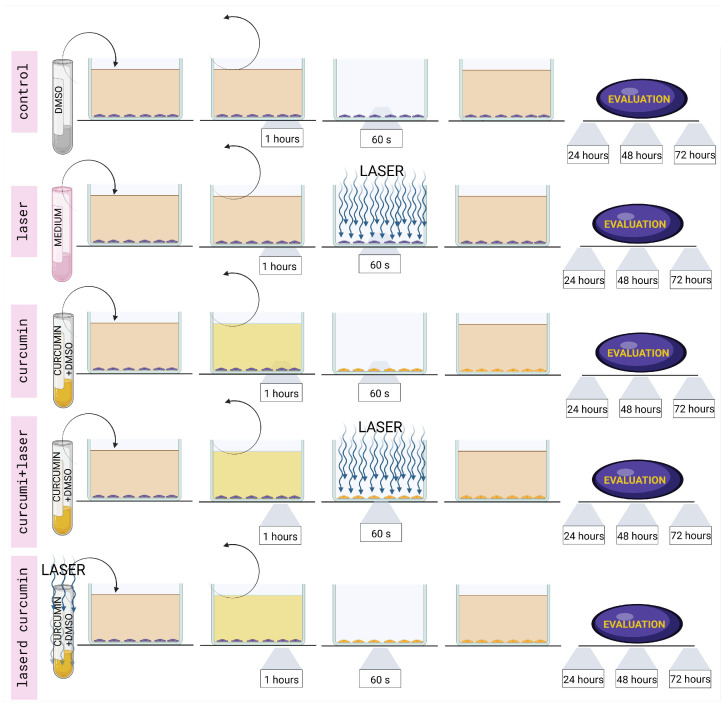
The experimental setup involved five conditions: (1) Untreated (Control): Cells were not exposed to either curcumin or laser. Cells were kept in DMEM supplemented with DMSO for one hour. The medium was removed, and the cells were not exposed to 450-nanometer diode laser irradiation. Fresh DMEM without curcumin was replaced. (2) Laser: An aliquot of DMEM medium equivalent to the volumes of curcumin used in other experiments was added to the cell culture’s DMEM. The cells were subsequently incubated in this medium for one hour. The medium was removed, and the cells were exposed to 450-nanometer diode laser irradiation. The supplemented DMEM was replaced with fresh DMEM without curcumin. (3) Curcumin: Cells were kept in a DMEM medium supplemented with curcumin in DMSO for one hour. The medium was then removed and replaced with fresh DMEM without curcumin. (4) Curcumin + laser: Cells were kept in DMEM supplemented with curcumin in DMSO for one hour. The medium was removed, and the cells were exposed to 450-nanometer diode laser irradiation. The supplemented DMEM was replaced with fresh DMEM without curcumin. (5) Lasered curcumin: Curcumin in DMSO was exposed to 450-nanometer diode laser irradiation before being added to supplement the DMEM medium. The cells were subsequently incubated in this medium for one hour. Afterward, the medium was removed and replaced with fresh DMEM without curcumin. The cells from all five conditions were placed in a dark incubator and monitored after 24, 48, and 72 h according to the experimental purposes.

In detail, the parameter of 15 J/cm^2^ and a time of irradiation of 60 s were selected because they are consistent with most studies on curcumin employment in photodynamic therapy for treating tumor cell lines. In addition, these parameters prevented there being any detrimental effects on the cells, as reported by Rocca et al. [23]. As illustrated in Figure 2, our experimental setup involved the five conditions described below:

Untreated (Control): Cells were not exposed to either curcumin or laser. They were kept in DMEM supplemented with DMSO for one hour. The medium was then removed, and the cells were not exposed to any 450-nanometer diode laser irradiation. The supplemented DMEM was replaced with fresh DMEM without curcumin.

Laser: An aliquot of DMEM medium equivalent to the volume of curcumin used in other experiments was added to the cell culture DMEM. The cells were subsequently incubated in this medium for one hour. The medium was then removed, and the cells were exposed to 450-nanometer diode laser irradiation. The supplemented DMEM was replaced with fresh DMEM without curcumin.

Curcumin: Cells were kept in DMEM medium supplemented with curcumin (0.1, 1, or 10 µM in DMSO) for 1 h. The medium was then removed and replaced with fresh DMEM without curcumin.

Curcumin + laser: Cells were kept in DMEM supplemented with curcumin (0.1, 1, or 10 µM in DMSO) for 1 h. The medium was then removed, and the cells were exposed to 450-nanometer diode laser irradiation. The supplemented DMEM was replaced with fresh DMEM without curcumin.

Lasered curcumin: Curcumin (0.1, 1, or 10 µM in DMSO) was exposed to 450-nanometer diode laser irradiation in solution before being added to the DMEM medium. The cells were subsequently incubated in this medium for one hour. Afterward, the medium was removed and replaced with fresh DMEM without curcumin.

The concentration of 10 μM was chosen because it represents a curcumin amount used in several in vitro treatments on cancer cell lines [12,24,25]. Lower concentrations were chosen to test whether irradiation rendered sublethal doses of curcumin more active against cancer cells, as proposed by Dukic et al. [26].

The five conditions were carried out under significantly reduced light conditions to prevent interference from natural light in the experiments. After treatment with one of the five conditions, cells were cultured for 72 h to assess cell growth, viability, and cell cycle every 24 h. Conversely, the biochemical experiments described below were all performed after 48 h of incubation. The absorption spectrum of UV and visible light (240–560 nm) of native curcumin was compared to that of the irradiated drug immediately after laser treatment T0) and after 1 h (T60) to assess whether irradiation with light at 450 nm causes the formation of curcumin degradation products. As expected, non-irradiated curcumin displayed a single absorption peak around 425 nm. Conversely, irradiated curcumin showed some less defined peaks between 280 and 350 nm, indicating the possible presence of curcumin degradation products. There were no significant differences in the peaks between irradiated curcumin evaluated at T0 and those evaluated at T60, suggesting that degradation products are formed instantaneously (Figure S1 in Supplementary Materials).

### 2.3. ATP Synthase Activity Assay

To measure the activity of ATP synthase, 20,000 cells were employed. The medium that the cells were resuspended in contained the following: 50 mM Tris-HCl (pH 7.4; #T1503, Merck, Darmstadt, Germany), 50 mM KCl (#P9333, Merck, Darmstadt, Germany), 1 mM EGTA (#03777, Merck, Darmstadt, Germany), 2 mM MgCl2 (#M2670, Merck, Darmstadt, Germany), 0.6 mM ouabain (#O0200000, Merck, Darmstadt, Germany), 0.25 mM di(adenosine)-5-penta-phosphate (an adenylate kinase inhibitor, #D1387, Merck, Darmstadt, Germany), and 25 μg/mL ampicillin (#A9393, Merck, Darmstadt, Germany). As respiration substrates, 10 mM pyruvate (#P4562 Merck, Darmstadt, Germany), 5 mM malate (#M8304 Merck, Darmstadt, Germany), and 0.1 mM ADP (#A5285, Merck, Darmstadt, Germany) were introduced following a permeabilization step with 0.03 mg/mL digitonin (#D5628, Merck, Darmstadt, Germany) for 1 min. Using a luminometer (GloMax^®^ 20/20n Luminometer, Promega Italia, Milan, Italy) and ATP standard solutions between 10-8 and 10-5 M, the luciferin/luciferase chemiluminescence method (luciferin/luciferase ATP bioluminescence assay kit CLS II, #11699695001 Roche, Basel, Switzerland) was used to monitor the reaction for 2 min, every 30 s [20].

### 2.4. Oxygen Consumption Assay

An amperometric electrode (Unisense Microrespiration, Unisense A/S, Aarhus, Denmark) was used to measure the amount of oxygen consumed. Twenty thousand cells were permeabilized in phosphate-buffered saline (PBS, #ECB4004, Euroclone, Pero (MI), Italy) for one minute using 0.03 mg/mL digitonin (#D5628, Merck, Darmstadt, Germany). To stimulate the route made up of Complexes I, III, and IV, 10 mM pyruvate (#P4562 Merck, Darmstadt, Germany), 5 mM malate (#M8304 Merck, Darmstadt, Germany), and 0.1 mM ADP (#A5285, Merck, Darmstadt, Germany) were added. To evaluate the OxPhos efficiency, the P/O ratio was calculated as the ratio between the quantity of the produced ATP and the amount of oxygen consumed. The P/O value is approximately 2.5 in normally functioning mitochondria when pyruvate and malate are present as respiration substrates, indicating that all oxygen consumption can be utilized to generate energy [27]. In contrast, when the P/O value is less than 2.5, part of the oxygen contributes to reactive oxygen species (ROS) generation rather than being used to produce energy.

### 2.5. Cell Homogenate Preparation

Cultured cells were detached with trypsin (#ECB3052, Euroclone, Pero (MI), Italy) and centrifuged at 1000 rpm for five minutes. The pellet was washed in PBS (#ECB4004, Euroclone, Pero (MI), Italy), resuspended in Milli-Q water, and sonicated twice for 10 s each in ice using a Microson XL Model DU-2000 (Misonix Inc., New Highway Farmingdale, NY, USA) device, with a 30 s break to prevent warming. The Bradford technique was used to estimate the total protein content [28].

### 2.6. Assay of Antioxidant Enzyme Activities

Catalase and glutathione reductase were chosen as examples of antioxidant enzyme activities as they represent the two main pathways through which oxidative stress detoxification occurs. In detail, catalases are part of the reactive oxygen species (ROS) detoxification pathway in concert with superoxide dismutase activity; glutathione reductase is part of a general cytosolic detoxification pathway that utilizes the reduced glutathione as a scavenger for all oxidized molecules, thereby including organic peroxides.

By measuring the oxidation of NADPH at 340 nm, the glutathione reductase (GR) activity was assayed spectrophotometrically. The assay mixture included the following components: 100 mM Tris HCl pH 7.4 (#T1503, Merck, Darmstadt, Germany), 1 mM EDTA #E9884, Merck, Darmstadt, Germany), 5 mM GSSH (#G4251, Merck, Darmstadt, Germany), and 0.2 mM NADPH (#N6505, Merck, Darmstadt, Germany).

By measuring the breakdown of H_2_O_2_ at 240 nm, the catalase (CAT) activity was assayed spectrophotometrically. The assay mixture contained 50 mM phosphate buffer pH 7.2 (#P3288, Merck, Darmstadt, Germany) and 5 mM H_2_O_2_ (#1.08597, Merck, Darmstadt, Germany). Data were normalized to the protein content of the sample (20 μg) for both assays.

### 2.7. Malondialdehyde Assay

Malondialdehyde (MDA) content was measured using the thiobarbituric acid reactive substances (TBARS) technique to investigate lipid peroxidation. This test is based on the interaction of thiobarbituric acid (TBA) and MDA, a breakdown product of lipid peroxides, to produce a colored product. The TBARS solution contained 26 mM thiobarbituric acid (#T5500, Merck, Darmstadt, Germany) and 15% trichloroacetic acid (TCA; #T9159, Merck, Darmstadt, Germany)) in 0.25 N HCl (#1.37007, Merck, Darmstadt, Germany). Then, 50 g of total protein was dissolved in 300 L of Milli-Q water, and 600 L of TBARS solution was added to determine the MDA basal concentration. At 95 °C, the mixture was incubated for 60 min. The sample was then centrifuged for two minutes at 14,000 rpm, and the supernatant absorbance was measured spectrophotometrically at 532 nm.

### 2.8. Evaluation of Cellular Growth and Cell Cycle

OHSU-974 cells were counted using a Burker chamber after trypan blue staining to evaluate the cellular growth rate. Cell viability was assessed by propidium iodide (PI) staining on unpermeabilized cells. Cell cycle phase distributions were determined from the DNA content histograms of at least 10 thousand cells. DNA content analysis was performed on cells permeabilized with 0.05% Triton X-100 and stained by flow-cytometric analysis, employing 30 mg/mL propidium iodide (PI) plus 0.5 mg/mL RNase for 30 min. Analyses were performed with a FACS-Calibur flow cytometer (Becton Dickinson, Franklin Lakes, NJ, USA).

### 2.9. Western Blot Analysis

We loaded 30 μg of proteins into a 4–20% gradient gel (#4561094, BioRad, Hercules, CA, USA) to perform a denaturing electrophoresis (SDS-PAGE). As primary antibodies, we employed anti-p53 (#9282, Cell Signaling Technology, Danvers, MA, USA), anti-CDK1 (#MA1-19057, Thermo Fisher Scientific, Waltham, MA, USA), and anti-Actin (#MA1-140, Thermo Fisher Scientific, Waltham, MA, USA), all diluted in PBS plus 0.15% Tween 20 (PBSt; #11332465001, Roche, Basel, Switzerland). A0168 and SAB3700870, both from Merck, Darmstadt, Germany, were used as specific secondary antibodies, and they were all diluted 1:10,000 in PBSt. Using a chemiluminescence system (Alliance 6.7 WL 20 M, UVITEC, Cambridge, UK) and an enhanced chemiluminescence substrate (ECL, # 1705061, BioRad, Hercules, CA, USA), bands were detected, and the signal intensity was measured by Uvitec-1D 2015 software (UVITEC, Cambridge, UK). The actin signal was used to normalize each band of interest.

### 2.10. Statistical Analysis

Tukey’s multiple comparison test was performed after a one-way analysis of variance (ANOVA) was used to analyze the data. The software utilized was Prism 8 (GraphPas software, Boston (MA), USA). The data are shown as mean ± standard deviation (SD) and are indicative of three or more independent experiments. Statistical significance was determined at a probability level of *p* < 0.05.

## 3. Result

### 3.1. Curcumin in Higher Doses Alone or with Laser Light Irradiation Produces a Decrease in Aerobic ATP Synthesis and an Increase in Oxygen Consumption by Inducing the Uncoupling of Oxidative Phosphorylation

**Figure 3:**
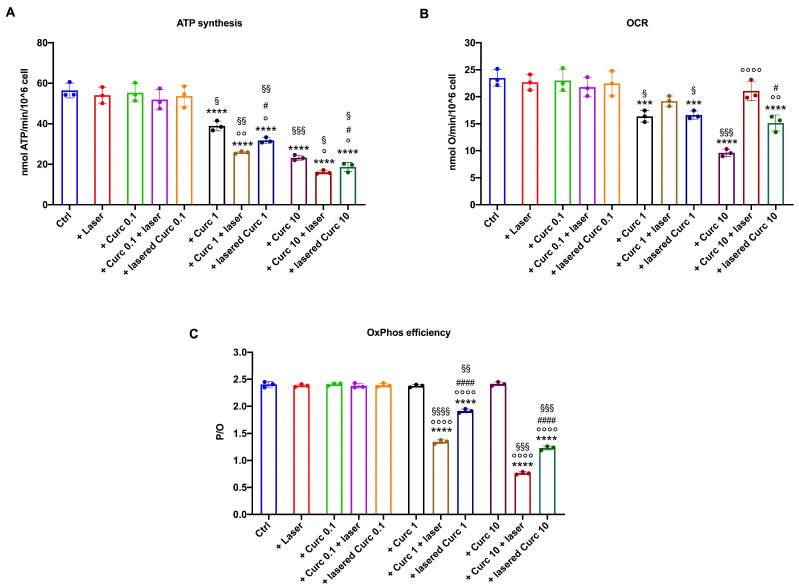
Effects of curcumin with or without irradiation on OHSU-974 OxPhos activity. (**A**) Aerobic ATP synthesis; (**B**) oxygen consumption rate (OCR); (**C**) P/O ratio, an OxPhos efficiency marker. Data are displayed as mean ± SD and are representative of 3 independent experiments. *** and **** indicate *p* < 0.001 or 0.0001, respectively, versus the control sample (untreated sample). °, °°, and °°°° indicate a *p* < 0.05, 0.01, or 0.0001, respectively, between samples treated with native curcumin and those treated with irradiated curcumin at the same concentration. # and #### indicate *p* < 0.05, 0.0001, respectively, between sample treated with curcumin irradiated after 1 h of incubation in the cells (Curc + laser) and lasered curcumin before administration to the cells (lasered Curc) at the same concentration. §, §§, §§§, and §§§§ indicate a *p* < 0.05, 0.01, 0.001, or 0.0001, respectively, between the different curcumin concentrations in the same treatment condition.

ATP synthesis and the oxygen consumption rate (OCR) were evaluated to investigate the effects of curcumin, in its native or irradiated form, on the energy metabolism of the OHSU-974 cell line. The data reported in Figure 3A show that the lowest concentration of curcumin tested (0.1 μM) and laser application alone did not affect OHSU-974 ATP synthase activity either in its native form or after irradiation. In contrast, 1 and 10 μM curcumin administered in its native form caused a significant decrease in aerobic ATP synthesis in OHSU-974 cells in a dose-dependent manner, which appeared to be further impaired when the cells were exposed to irradiated curcumin. However, it is interesting to note that the curcumin’s effect appeared to differ based on the timing of irradiation: the treatment was more effective in inhibiting ATP synthesis when the curcumin was incubated for one hour in OHSU-974 cells, which were then subjected to laser treatment (Curc + laser), rather than when the drug was irradiated before its administration to OHSU-974 (lasered Curc, Verona, Italy).

Like ATP synthesis, the laser alone and the lowest curcumin concentration did not cause changes in the oxygen consumption rate (OCR) compared to the control (Figure 3B). In contrast, 1 and 10 μM curcumin administrated alone inhibited respiration in a dose-dependent manner, whereas, when irradiated, it induced an increase in the OCR compared to native curcumin (Figure 3B). This biphasic behavior suggests that curcumin irradiation causes an imbalance between the electron transport chain and ATP synthase. This hypothesis was confirmed by the evaluation of the P/O ratio, a marker of OxPhos efficiency, which decreased markedly only when curcumin was irradiated, reaching its maximum decrease when the maximum curcumin concentration (10 μM) was lasered after 1 h of incubation in OHSU-974 cells (Figure 3C). In other words, curcumin in the dark proportionally decreased both ATP synthesis and respiration, leaving the residual activity coupled; conversely, irradiation inhibited ATP synthase activity (despite an increase in OCR) and induced OxPhos uncoupling.

### 3.2. Curcumin in Higher Doses Alone or with Laser Light Irradiation Causes a Further Decrease in Cellular Energetics Compared to the Native Molecule

**Figure 4:**
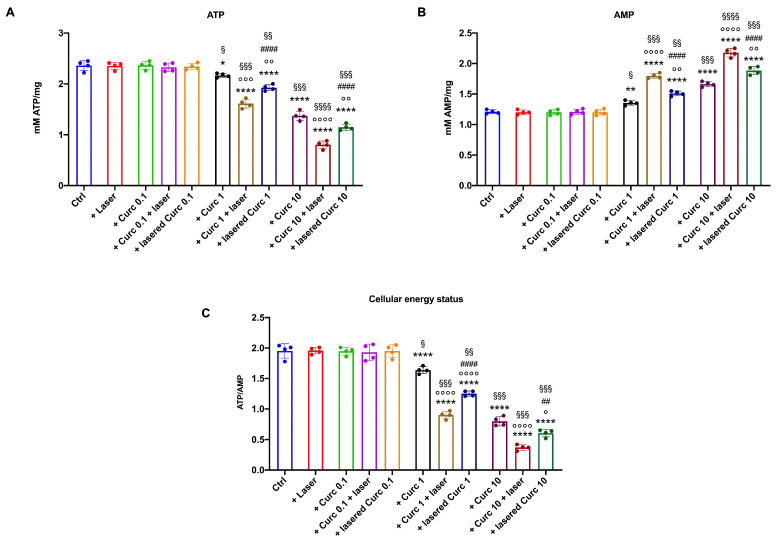
Irradiated curcumin effect on OHSU-974 energy status. (**A**) ATP intracellular concentration; (**B**) AMP intracellular concentration; (**C**) ATP/AMP ratio, a marker of cellular energy status. Data are displayed as mean ± SD and are representative of 4 independent experiments. *, **, **** indicate *p* < 0.05, 0.01, or 0.0001, respectively, versus the control sample (untreated sample). °, °°, °°°, and °°°° indicate *p* < 0.05, 0.01, 0.001, or 0.0001, respectively, between samples treated with native curcumin and those treated with irradiated curcumin at the same concentration. ## and #### indicate *p* < 0.01, 0.0001, respectively, between the sample treated with curcumin irradiated after 1 h of incubation on the cells (Curc + laser) and lasered curcumin before its administration on the cells (lasered Curc) at the same concentration. §, §§, §§§, and §§§§ indicate *p* < 0.05, 0.01, 0.001, or 0.0001, respectively, between the different curcumin concentrations in the same condition.

To investigate whether the changes in OxPhos may affect the OHSU-974 energetic state, the ATP/AMP ratio was evaluated by measuring intracellular ATP and AMP concentrations. The data showed a similar trend to that observed for aerobic ATP synthesis: the laser alone and the lowest concentration of curcumin tested caused no change in the intracellular concentration of ATP or AMP compared to the control (Figure 4A and Figure 4B, respectively). On the contrary, both 1 and 10 μM curcumin, even under dark conditions, caused a decrease in ATP (Figure 4A) and an increase in AMP (Figure 4B). These effects were amplified when the molecule was irradiated (Figure 4A,B). Like the OxPhos activity data, the most evident effect was obtained when OHSU-974 cells were incubated with 10 μM curcumin for 1 h before laser treatment. This effect may be because (i) curcumin induces OxPhos uncoupling as well as the ATP synthase inhibition, and (ii) a stressed system requires a higher energy demand to maintain homeostasis.

The ATP/AMP ratio was lowered due to the low ATP and high AMP intracellular levels in OHSU-974 cells treated with 1 and 10 μM curcumin (Figure 4C).

### 3.3. Curcumin in Higher Doses Alone or with Laser Light Irradiation Induces an Antioxidant Response That Is Insufficient to Limit Oxidative Damage

**Figure 5:**
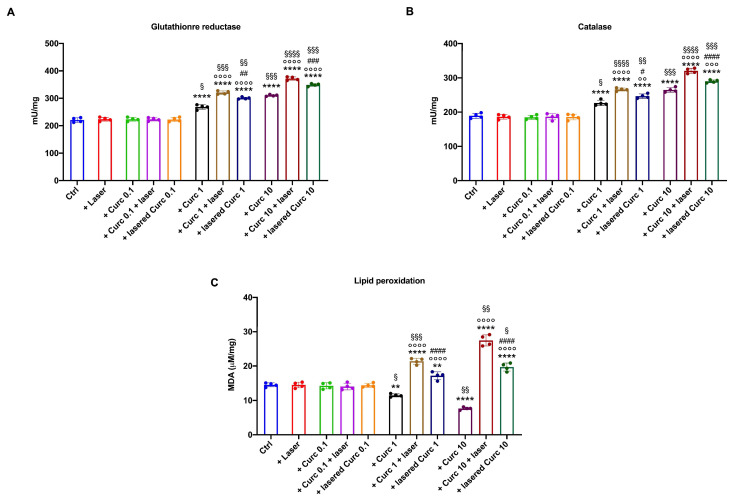
Effects of irradiated or non-irradiated curcumin on OHSU-974 enzymatic antioxidant defenses and lipid peroxide accumulation. (**A**) Glutathione reductase activity; (**B**) catalase activity; (**C**) malondialdehyde content, a lipid peroxidation marker. Data are displayed as mean ± SD and are representative of 4 independent experiments. ** and **** indicate *p* < 0.01 or 0.0001, respectively, versus the control sample (untreated sample). °°, °°° and °°°° indicate *p* < 0.01, 0.001 or 0.0001, respectively, between samples treated with native curcumin or with irradiated curcumin at the same concentration. #, ##, ### and #### indicate *p* < 0.05, 0.01, 0.001, or 0.0001, respectively, between the sample treated with curcumin irradiated after 1 h of incubation in the cells (Curc + laser) and lasered curcumin before the administration to the cells (lasered Curc) at the same concentration. §, §§, §§§, §§§§ indicate *p* < 0.05, 0.01, 0.001, or 0.0001, respectively, between the different curcumin concentrations in the same treatment condition.

Since curcumin can positively modulate the cellular antioxidant defenses [25], the activity of glutathione reductase (GR) and catalase (CAT), two enzymes belonging to distinct pathways involved in reactive oxygen species detoxification, were measured. The data show that curcumin in its native form at 1 and 10 μM increased GR (Figure 5A) and CAT (Figure 5B) activity. Due to the increased antioxidant defenses and the slowing down of OxPhos caused by curcumin in the dark, a decrease in malondialdehyde (MDA) accumulation (Figure 5C) was observed.

Interestingly, a further increase in GR and CAT activity was observed when curcumin was irradiated (Figure 5A,B). However, this increase did not appear sufficient to counterbalance the rise in oxidative stress due to OxPhos uncoupling because malondialdehyde accumulation was observed in OHSU-974 after curcumin irradiation. The MDA levels showed a trend proportional to the OxPhos uncoupling (Figure 5C) and were more evident when curcumin was irradiated after cell administration. Also, in this case, a higher effect was observed with 10 μM laser-treated curcumin after one hour of incubation in the cells. Conversely, laser alone and low-dose curcumin (0.1 μM) did not cause any increase in antioxidant response or lipid peroxidation compared to the control.

### 3.4. Curcumin in Higher Doses Alone or with Laser Light Irradiation Slows Cell Growth, Decreases Cell Viability, and Blocks the Cell Cycle at the S-G2M Phase Compared to Curcumin in the Dark

**Figure 6:**
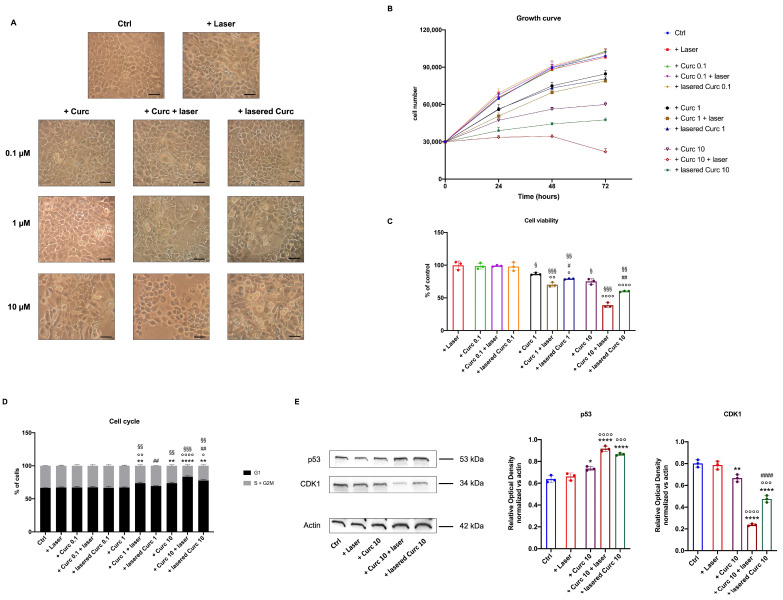
Effects of irradiated curcumin on OHSU-974 morphology, cell growth, viability, and cell cycle. (**A**) Example of OHSU-974 morphology observed by transmitted-light microscopy. The scale bar corresponds to 100 μm. Each panel is representative of at least 4 fields from 4 independent experiments; (**B**) OHSU-974 growth curve; (**C**) percentage of cell viability compared to the control samples; (**D**) proliferative response of OHSU-974; (**E**) Western blot signals of the expression of p53 and CDK1, markers of G1 and G2/M phase, respectively, and related signals densitometric analysis normalized versus actin signal, used as a housekeeping protein. For panels (**B**–**D**), and histograms in (**E**), data are reported as mean ± SD, and each graph is representative of 3 independent experiments. The uncropped blots are shown in Figure S2. *, **, and **** indicate *p* < 0.05, 0.01, or 0.0001, respectively, versus the control sample (untreated sample). °, °°, °°°, °°°° indicate *p* < 0.05, 0.01, 0.001, or 0.0001, respectively, between samples treated with native curcumin and those treated irradiated curcumin at the same concentration. #, ##, and #### indicate *p* < 0.05, 0.01, or 0.0001, respectively, between the sample treated with curcumin irradiated after 1 h of incubation in the cells (Curc + laser) and lasered curcumin before administration to the cells (lasered Curc) at the same concentration. §, §§, §§§ indicate *p* < 0.05, 0.01, or 0.001, respectively, between the different curcumin concentrations in the same treatment condition.

The cellular growth and cycle were evaluated to investigate whether changes in energy metabolism due to irradiated curcumin caused any effect on OHSU-974 cell biology. The data in Figure 6A show a morphological change in the cells as the concentration of curcumin increases, which becomes even more evident when the drug is irradiated, especially after incubation on the cells. A slowdown in cell growth (Figure 6B) and decreased cell viability (Figure 6C) after treatment with 1 and 10 μM curcumin was observed, which further increased when the molecule was laser-treated, reaching a minimum when cells were lasered after treatment with 10 μM curcumin for 1 h (Figure 6B and Figure 6C, respectively). Furthermore, irradiated curcumin caused a cell cycle arrest at the S-G2M phases (Figure 6D). In addition, the expression of p53 (a G1 phase marker) and CDK1 (a G2/M phase marker) was assessed (Figure 6E) to confirm the cytofluorimetric analysis of the cell cycle. The data show that while laser treatment alone did not change the expression of p53 and CDK1, treatment with native curcumin alone caused a slight but significant increase in p53 and a lowering of CDK1, a trend most evident when curcumin was irradiated, especially when the drug was lasered after one hour of incubation on the cells.

### 3.5. Curcumin in Higher Doses Alone or with Laser Light Irradiation Causes a Decrease in Energy Status and Growth and an Increase in Lipid Peroxidation Accumulation in Primary Human Fibroblasts

**Figure 7:**
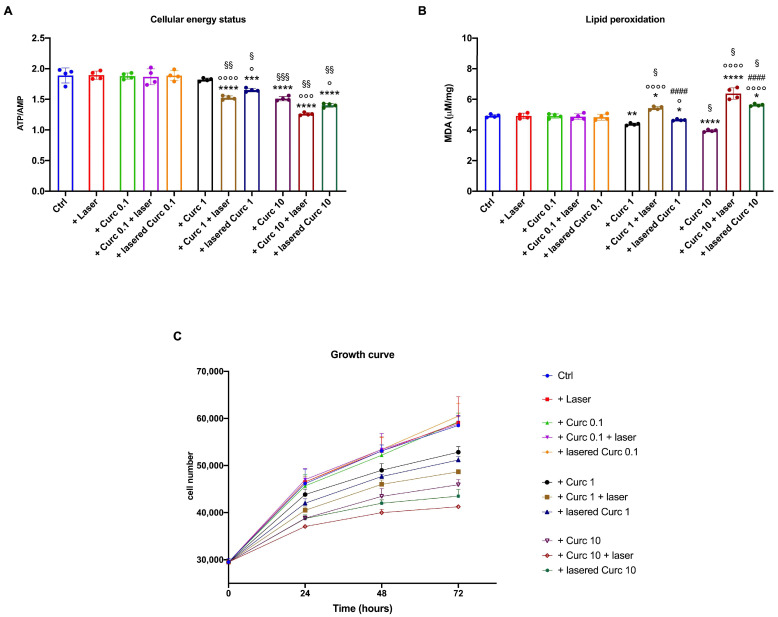
Effects of irradiated curcumin on primary human fibroblasts energy status, lipid peroxidation, and cell growth. (**A**) ATP/AMP ratio, a marker of cellular energy status; (**B**) malondialdehyde content, a lipid peroxidation marker; (**C**) growth curve. Data are represented as mean ± SD and are representative of 4 independent experiments. *, **, ***, **** indicate *p* < 0.05, 0.01, 0.001, or 0.0001, respectively, versus the control sample (untreated sample). °, °°°, °°°° indicate *p* < 0.05, 0.001, or 0.0001, respectively, between samples treated with native curcumin and those treated with irradiated curcumin at the same concentration. #### indicate *p* < 0.0001 between the sample treated with curcumin irradiated after 1 h of incubation in the cells (Curc + laser) and lasered curcumin before the administration to the cells (lasered Curc) at the same concentration. §, §§, and §§§ indicate *p* < 0.05, 0.01, or 0.001, respectively, between the different curcumin concentrations in the same treatment condition.

Since fibroblasts play a pivotal role in the maintenance of the HNSCC microenvironment, the effect of curcumin, irradiated or not, was evaluated on primary human fibroblasts. As for OHSU-974, the lower curcumin dose (0.1 μM) and laser alone did not cause modifications in the cellular energy status (Figure 7A), lipid peroxidation accumulation (Figure 7B), or cell growth (Figure 7C). By contrast, fibroblasts treated with 1 and 10 μM curcumin showed a decrement in the ATP/AMP ratio, a reduction in lipid peroxidation, and a slight but significant slowdown in cell growth in a dose-dependent manner. More detrimental effects were observed after curcumin irradiation (especially after cell incubation), which, in addition to causing further damage to metabolism and cell growth, also induced an accumulation of malondialdehyde. However, although the effects on human fibroblasts were similar to those observed on OHSU-974, their magnitude appears to be lower.

## 4. Discussion

The presented data indicate that curcumin inhibits ATP synthesis and the OCR in OHSU-974 cells in a dose-dependent manner, starting from a concentration of 1 µM administered in the dark. The concentration of 0.1 µM did not affect the energy metabolism, while 10 µM had a stronger effect. The proportional decrease in ATP synthesis and the OCR indicated that the residual activity remained coupled. In other words, in its native form, curcumin exerts its anti-cancer effect by decreasing OxPhos activity and the resulting production of oxidative stress, which are two essential building blocks for the rapid growth of cancer cells [29]. These data reflect the role of curcumin as an antioxidant molecule that can work by inhibiting ATP synthesis and, therefore, reducing oxygen consumption in the coupled mitochondrial machinery. Subsequently, the production of reactive oxygen species and the relative oxidative damage are reduced [30]. After all, in a previous in vitro study performed on L1210 murine lymphocytic leukemia, 4T1 murine breast, B16 murine melanoma, and CT26 murine colon tumor cell lines, it was found that 10 µM curcumin could inhibit the activity of ATP synthase in isolated mitochondrial membranes, resulting in a dramatic drop in ATP and a reduction in oxygen consumption [12]. Moreover, our data show that curcumin alone at 1 and 10 µM increases GR and CAT activity, and our data are consistent with the literature showing that curcumin positively modulates antioxidant responses [31]. Because of this enhancement of antioxidant defenses and the slowing down of OxPhos, a decrease in oxidative stress and damage accumulation is observed in OHSU-974 cells. The decrease in energy metabolism resulted in morphological changes in the cells, a decrease in cell growth, and a decrease in cell viability after being treated with 1 and 10 μM curcumin. On the other hand, the literature reports that curcumin has dose-dependent activity in treating the A549 human lung cancer cell line; doses of 20 and 40 µM lead to the downregulation of Nuclear Factor-kappa B (NF-kB) [32]. In addition, all of the tested curcumin concentrations decreased cell viability and induced apoptosis in human non-small cell lung cancer cells in a dose- and time-dependent manner, with the treatment time ranging between 12, 24, and 48 h. This effect was achieved by the upregulation of microRNA-192-5p and suppression of the phosphoinositide 3-kinase/protein kinase B (PI3K/Akt) signaling pathway [33]. In addition, a cationic lipid nanosystem incorporating 20.25 μM and 39.70 μM curcumin demonstrated enhanced cytotoxicity against Lewis’ lung cancer cells, exhibiting increased antiproliferative, proapoptotic, and anti-invasive activity, as well as inducing cell cycle arrest [34].

In vitro studies on head and neck cancer, mainly squamous cell carcinoma, have described apoptosis of cell lines following treatment with curcumin in a range from 3 to 400 µM, depending on the cell line [35]. This was achieved through direct effects involving pro-apoptotic and anti-apoptotic gene expression and indirect effects such as G2/M cell cycle arrest and an increase in the sub-G1 cell population. Curcumin could act on various cellular targets, including NF-κB, cyclin D1, B-cell lymphoma 2 (Bcl-2), nitric oxide synthase (NOS), cyclooxygenase-2 (COX-2), interleukins, tumor necrosis factor-alpha (TNF-α), and matrix metalloproteinase-9 (MMP-9). According to a review by Borges et al. [35], only the Detroit 562 cells showed an IC50 as low as 3 µM. Conversely, some other cell lines were not affected by 0.1–5 µM of curcumin or its metabolic derivatives but were affected by a moderate concentration of 10 µM or higher. Overall, the data suggest that HNSCC cell lines are sensitive to low-to-moderate curcumin concentrations, which could facilitate the translation of the results into in vivo experimentation. In fact, despite its potential anti-cancer activity in vitro, translating this approach to in vivo efficacy is still a challenge due to the high doses required [8] because curcumin has poor absorption, rapid metabolism, and other bioavailability problems [14]. A study conducted on rats revealed that when curcumin was taken orally (1 g/kg), 75% of it was excreted in urine rather than absorbed by the digestive system [36]. After taking 400 mg of curcumin by mouth, 90% was found in the small intestine and stomach after 30 min, but only 1% was present after 24 h. Other studies have demonstrated that curcumin metabolites may be less active than curcumin itself [37]. Furthermore, the administration of high concentrations of curcumin, although not toxic, can potentially be hepatotoxic and lead to side effects such as nausea, diarrhea, headaches, and the discoloration of stools to a yellowish hue [38]. Thus, the limits of the therapeutic use of curcumin in vivo might be overcome by its enhanced efficacy on OHSU-974 cells after activation with 450 nm blue light.

Our data indicate that irradiation of curcumin leads to OxPhos uncoupling, increasing the production of oxidative stress, as demonstrated by elevated oxygen consumption and MDA accumulation, along with a reduction in ATP synthase activity. Interestingly, the effects were more pronounced when curcumin was irradiated after cell administration than before. Metabolic changes that occur when curcumin is internalized into cells and photoactivation followed by the photodynamic effect or photodegradation may impact this difference. The literature reports that cells rapidly metabolize curcumin, mainly by reduction and conjugation [39,40], forming new chemical species that, once exposed to light, could be more effective in altering energy metabolism.

Theoretical calculations conducted by Shen and Ji [11] reported that the enolic group of curcumin was more affected by visible light than the phenolic group, and the enolic form prevails in solution with maximum absorption in the wavelength range we used. The authors suggested that the enol form of curcumin had the highest probability of being effective on tumors [11]. These mechanisms and the presence of some curcumin, which may remain unmetabolized within 1 h, could simultaneously occur within cells and affect the photodynamic therapy.

On the other hand, visible light and UV rays can affect the biological properties of curcumin because of the fragmentation of the dicarbonyl system leading to the formation of degradation products such as vanillin, ferulic acid, vanillic acid, and ferulaldehyde [41]. Our study highlights that curcumin exhibits antitumor properties whose intensity or mode varies depending on the type of administration or treatment (in dark mode, pre-irradiated, or photoactivated post-administration). This knowledge can assist the decision to use various therapeutic approaches that complement existing treatments. Based on the literature review, little attention has been devoted to photodynamic curcumin therapy for human HNSCC. Among these reports, Beyer and colleagues [4] demonstrated that the pre-incubation of human HNSCC cell lines for 1 h with curcumin concentrations ranging from 0.027 μM to 2.71 μM, followed by exposure to either 1 J/cm^2^ UVA or a Philips visible light bulb (spectrum: 380–780-nm) for 5 min, resulted in a reduction in cell proliferation and increased reactive oxygen species generation while increasing DNA fragmentation. Another study by Dujic and colleagues [42] demonstrated that when cells were pre-treated with curcumin concentrations ranging from 0.677 μM to 5.42 μM for 1 h, followed by exposure to either UVA (1 J/cm^2^) or a Philips visible light bulb (5500 l×, spectrum: 400–500 nm) for 5 min, an intriguing result was observed. Specifically, the combination of 2.71 μM curcumin with visible light led to a significant reduction in proliferation down to 17.3%, whereas the reduction achieved with the UVA combination was only 31.1%. Additionally, a decrease in the metabolic rate was observed in cells treated with curcumin and light but not in the absence of light. Notably, the combination with visible light exhibited a more pronounced decrease in cell viability compared to UVA, resulting in a reduction of 50% and 35%, respectively.

Our findings are consistent with prior studies, indicating a more pronounced effect from combination therapy. When comparing similar incubation times, it appears that 450 nm wavelength laser light source yields superior outcomes after just 60 s of irradiation compared to broad-spectrum light produced by a bulb due to the photodynamic or photodegradative action of 450 nm light on curcumin rather than photobiomodulation (the process of transferring photon energy to the cell that accelerated healing and increased mitochondrial efficiency). As photoacceptors, flavins can absorb blue light, with flavin adenine dinucleotide (FAD) and flavin mononucleotide (FMN) playing roles in the respiratory chain’s electron transport. However, our light-alone parameters did not impact the analyzed cells. Cytotoxic effects by irradiated curcumin were also observed in normal human fibroblasts, which play a pivotal role in the head-neck tumor microenvironment [17,18], although they appeared to be less severe. In detail, in the most cytotoxic conditions (10 µM photo-activated curcumin), we observed an 18% reduction in the cellular energetic state, contrasting with the 80% reduction observed in the HNSCC model. It is likely that the accumulation of MDA was slower, and there was a mere slowdown in fibroblast cell growth as opposed to the evident cell death seen in OHSU-974 cells. This different behavior probably depends on the lower metabolic rate of fibroblasts compared to HNSCC cells. On the other hand, some studies have reported the production of blue light-induced reactive oxygen species in gingival fibroblasts’ mitochondria and mitochondrial DNA damage in epithelial cells [43].

## 5. Conclusions

In conclusion, curcumin shows an ability to reduce the aerobic energy metabolism function of HNSCC at concentrations of 1 and 10 μM. The anti-tumor activity of curcumin was further enhanced by a 60 s exposure to 15 J/cm^2^ of 450 nm laser light, underscoring the significance of identifying therapeutic concentrations that are both low and effective. This becomes pivotal considering the constrained bioavailability of curcumin following oral or intravenous administration. A noteworthy aspect of our study is the discovery that native curcumin and its irradiated counterpart induce an anti-tumor effect by acting similarly on energy metabolism but differently on oxidative stress production. The natural, photodegraded, or photoactivated state of the molecule strongly could support the curcumin’s behavior. Finally, although photodynamic therapy using visible light may be somewhat limited in its ability to penetrate tissues and reach the target in vivo, the nature of the tumor we analyzed, which develops from squamous cells typically found in the outermost layers of the skin and mucous membranes, appears to be promising even in vivo. In particular, this seems to hold in the early stages when the tumor has not yet extended deeply into the surrounding tissues. To bolster the credibility of our findings, in vivo studies are imperative. Given the unique characteristics of HNSCC, investigating alternative administration methods of curcumin and laser light, such as subcutaneous injection/irradiation or nanostructures, holds promise for overcoming systemic bioavailability limitations, facilitating sustained molecule release, and improving light tissue penetration.

## Data Availability

Data are available on request from the authors.

## Supplementary Materials

The following supporting information can be downloaded at: https://www.mdpi.com/article/10.3390/cancers16091642/s1, Figure S1: Absorption of UV and visible spectra of native and irradiated curcumin. Data are expressed as the mean of three independent experiments. T0 indicates the curcumin evaluated immediately after the irradiation. T60 min indicates the curcumin evaluated one hour after the irradiation. Figure S2: Uncropped signals of western blot reported in Figure 6. Above each band, the densitometric value is reported.
