# Supplementary material for: Assessing the Effects of Curcumin and 450 nm Photodynamic Therapy on Oxidative Metabolism and Cell Cycle in Head and Neck Squamous Cell Carcinoma: An In Vitro Study
Source: Cancers (Basel). 2024 Apr 24;16(9):1642. doi: 10.3390/cancers16091642 (PMC11083672; doi:10.3390/cancers16091642)
Supplement: Supplementary file 1 [file cancers-16-01642-s001.zip › cancers-2957521-supplementary.pdf]

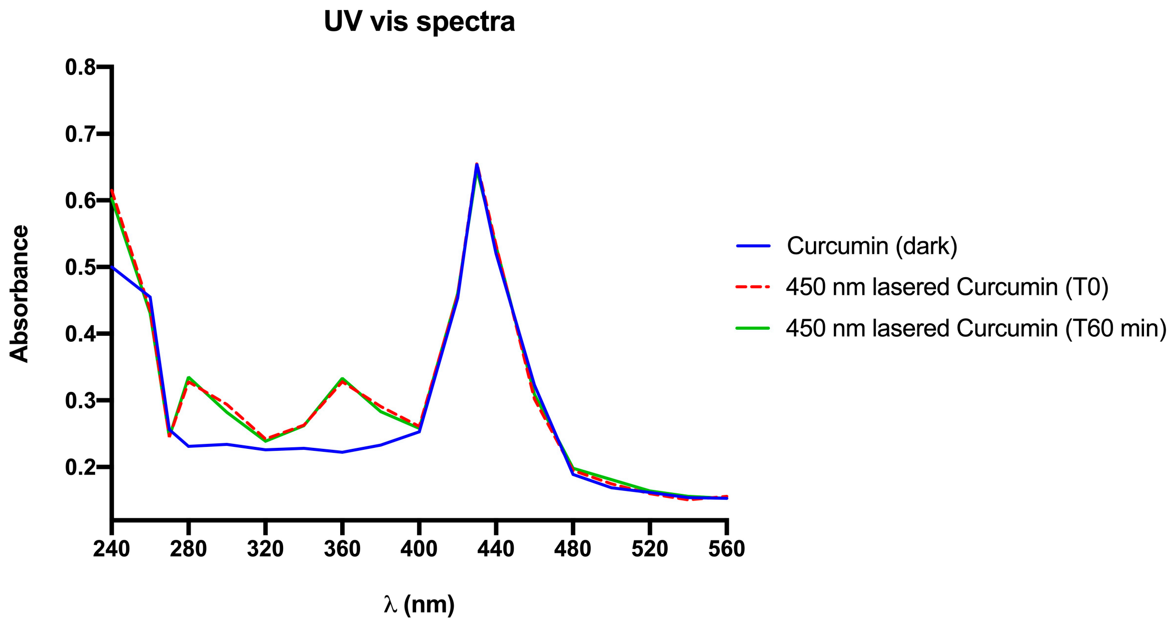

Figure S1. Absorption of UV and visible spectra of native and irradiated curcumin. Data are expressed as the mean of three independent experiments. T0 indicates the curcumin evaluated immediately after the irradiation. T60 min indicates the curcumin evaluated one hour after the irradiation.

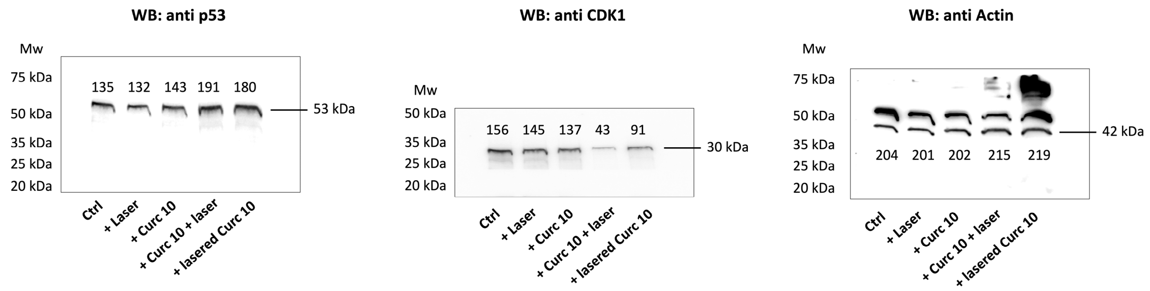

Figure S2. Uncropped signals of western blot reported in Figure 6. Above each band, the densitometric value is reported.
